# Bex-Nikaidoh Procedure for Complex Transposition of Great Arteries With Ventricular Septal Defect and Left Ventricular Outflow Tract Obstruction: A Case Report

**DOI:** 10.7759/cureus.80216

**Published:** 2025-03-07

**Authors:** Pheng Hian Tan, Ahmad Nazrin Ja'apar, Mathan Mohan Munusamy, Ahmad Sallehuddin

**Affiliations:** 1 Cardiothoracic Surgery, National Heart Institute, Kuala Lumpur, MYS; 2 Pediatric Cardiology, National Heart Institute, Kuala Lumpur, MYS

**Keywords:** aortic root translocation, bex-nikaidoh, pulmonary stenosis, transposition of great arteries, ventricular septal defect

## Abstract

Several surgical approaches have been proposed to correct the transposition of the great arteries (TGA) with ventricular septal defect (VSD) and left ventricular outflow tract obstruction (LVOTO). Unlike the traditional procedures such as the Rastelli and Réparation à l'Etage Ventriculaire (REV) procedure, which often necessitate reoperation due to right or left ventricular outflow tract obstruction, the Bex-Nikaidoh procedure involves the translocation of both the aortic and pulmonary roots to achieve anatomically correct outflow alignment for both ventricles. Although technically demanding, this technique effectively addresses the anatomical challenges such as inlet or restrictive VSD and hypoplastic right ventricle. Recent studies indicate encouraging mid-term results, including high survival rate, preserved left ventricular function, and lower incidence of reoperation. We present a case report of a child with TGA, VSD, and pulmonary stenosis who successfully underwent the Bex-Nikaidoh procedure, detailing our operative technique, perioperative assessment, and management, thereby contributing to the evolving landscape of surgical repair for complex TGA with VSD and LVOTO.

## Introduction

The combination of transposition of great arteries (TGA) with ventricular septal defect (VSD) and left ventricular outflow tract obstruction (LVOTO) inhibits arterial switch operation (ASO), making optimal surgical management challenging. Several surgical techniques have been proposed. The Rastelli procedure, introduced in 1969, involves an intracardiac baffle that tunnels the left ventricle to the aorta and an extracardiac valved conduit connecting the right ventricle to the pulmonary artery [1]. The Réparation à l’Etage Ventriculaire (REV) procedure, introduced in 1980, restores right ventricle to pulmonary artery continuity without an extracardiac conduit and reduces the risk of right ventricular outflow tract obstruction (RVOTO) [2]. However, both the Rastelli and REV procedures demonstrate significant limitations in optimizing the ventricular outflow tract flow dynamics as both techniques exhibit abnormal flow patterns in the left ventricular outflow tract, and the Rastelli procedure is associated with a high incidence of RVOTO requiring reoperation [3].

The Bex-Nikaidoh procedure, introduced in 1984, involves translocating both the aortic and pulmonary roots [4]. This technique results in a more anatomical alignment of the right and left ventricular outflow tracts, reducing the risk of both RVOTO and LVOTO. The Bex-Nikaidoh procedure is particularly advantageous in cases with inlet or restrictive VSD and hypoplastic right ventricle [5]. Despite its technical complexity and longer operative time, recent studies have shown encouraging mid-term results with excellent survival rate, preserved left ventricular function, and lower incidence of reoperation [6]. This case report describes our experience in treating a child with TGA, VSD, and pulmonary stenosis (PS) using the Bex-Nikaidoh procedure, highlighting its potential benefits in addressing this challenging congenital heart defect.

## Case presentation

**Figure 1:**
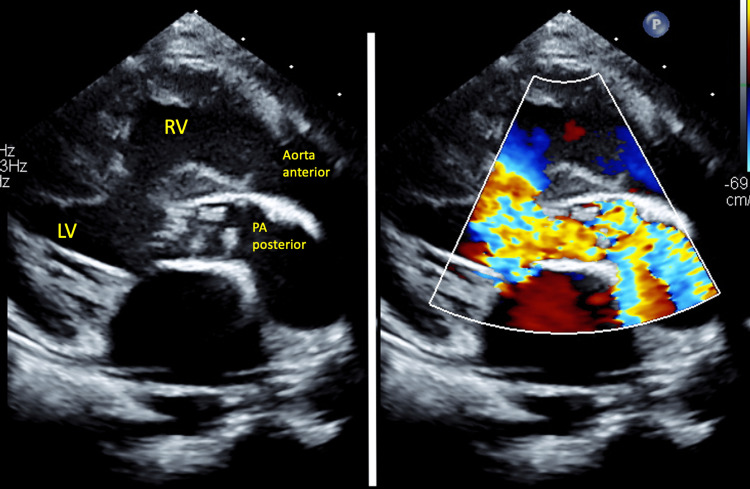
Preoperative echocardiogram (parasternal long axis view) showing the relation of the aorta to the pulmonary artery LV, left ventricle; PA, pulmonary artery; RV, right ventricle

A non-syndromic one-year seven-month-old girl was diagnosed with TGA, VSD, and PS (TGA/VSD/PS) since birth. She underwent balloon atrial septostomy on day 3 of life. She also has severe right sensorineural hearing loss with speech delay. She presented with progressive cyanosis and was planned for the Bex-Nikaidoh procedure. Her peripheral oxygen saturation (SPO2) was 77%, and her body weight was 8.6 kg. Preoperative echocardiogram (Figure 1) showed d-TGA with balanced ventricles, left ventricular ejection fraction (EF) of 73%, a 9mm muscular outlet to mid-muscular VSD, PS with pulmonary annulus of 7-8mm, and a mean pressure gradient of 49mmHg. Her baseline hemoglobin was 17.0g/dL, white blood count was 13.6 x 10^9^/L, and platelet count was 319 x 10^9^/L. Her baseline renal and liver profiles were both normal.

**Figure 2:**
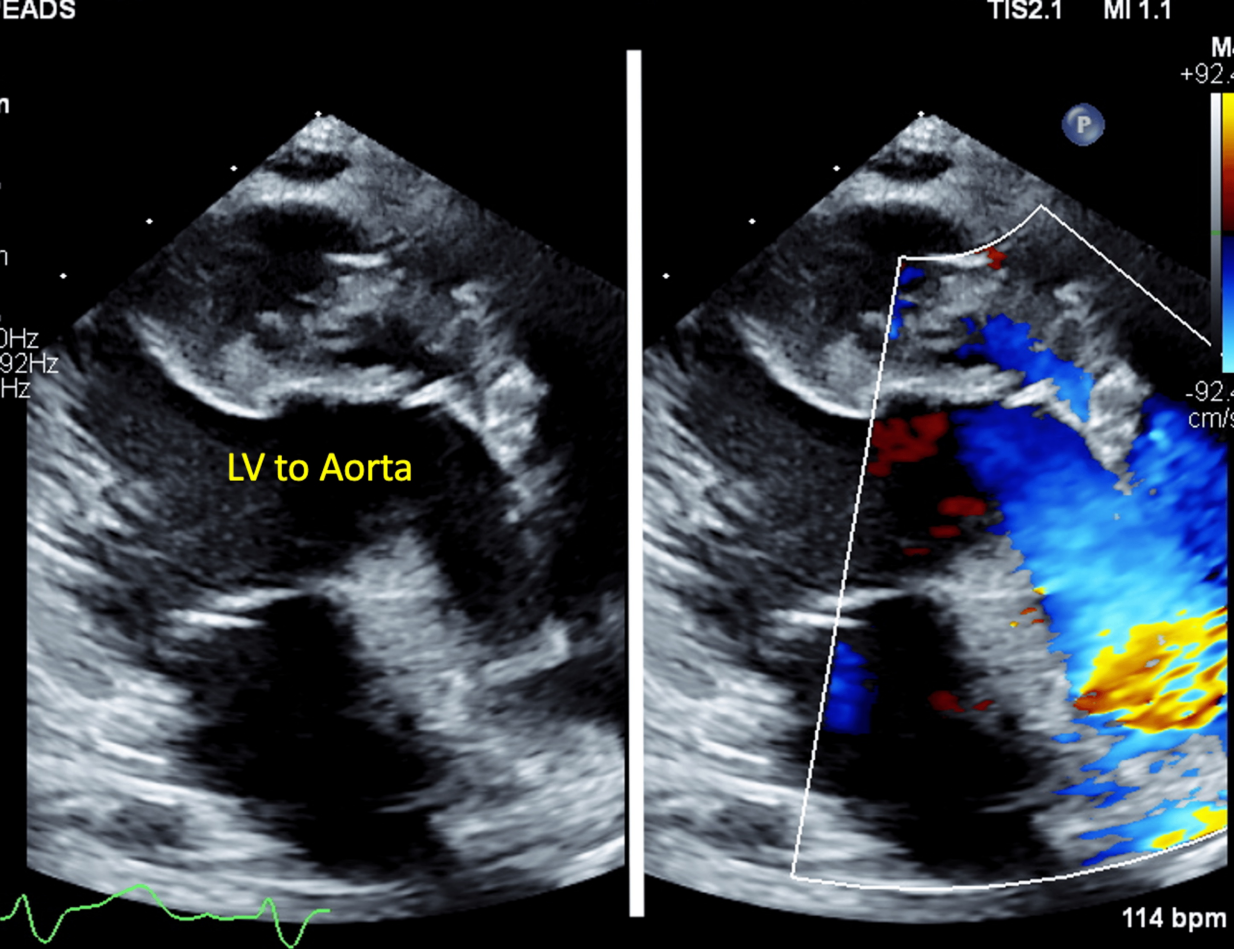
Postoperative echocardiogram (parasternal long axis view) showing the patent left ventricular outflow tract LV, left ventricle

**Figure 3:**
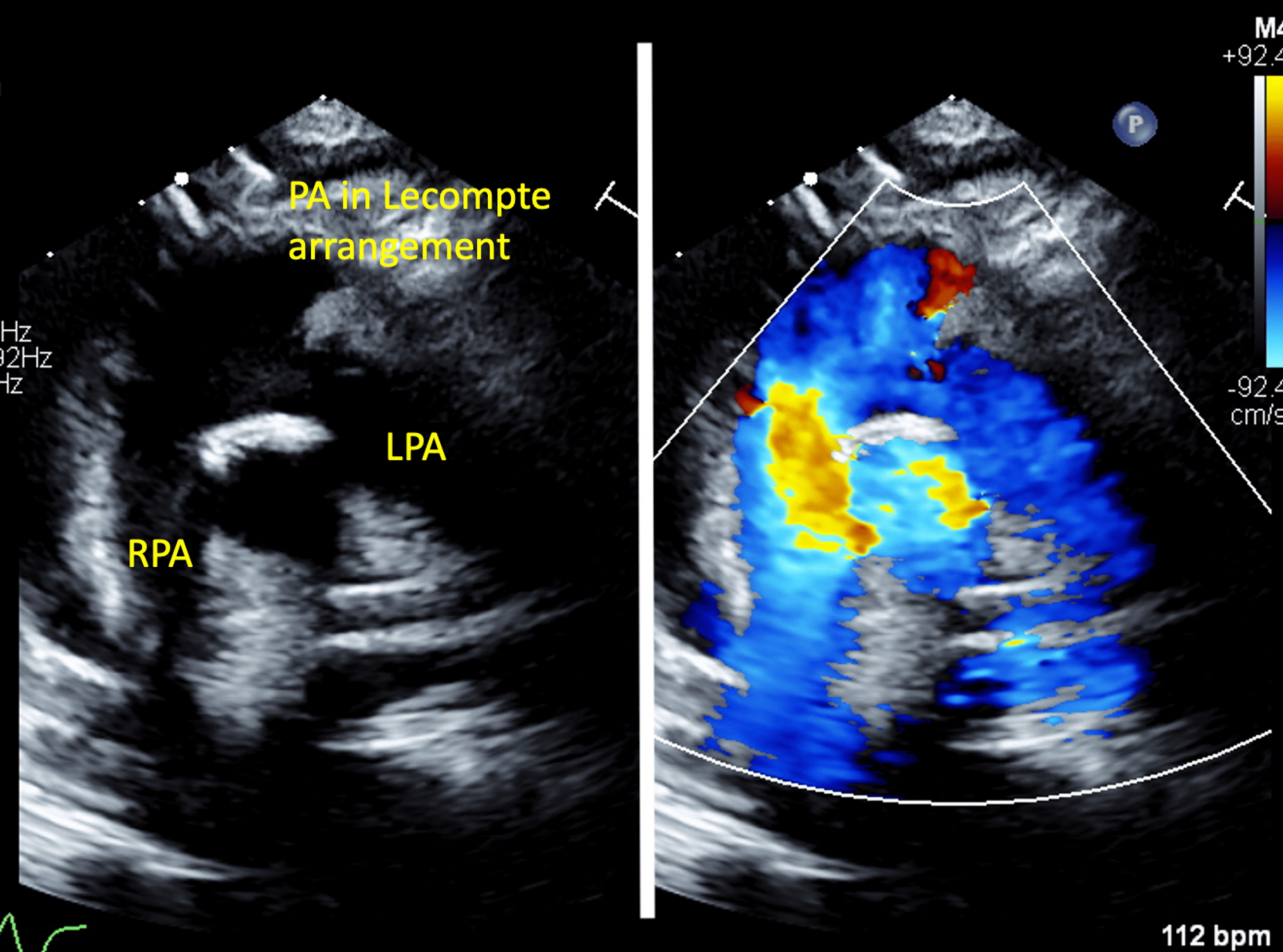
Postoperative echocardiogram (modified suprasternal view) showing the patent right ventricular outflow tract LPA, left pulmonary artery; PA, pulmonary artery; RPA, right pulmonary artery

The operation was performed successfully. The cardiopulmonary bypass time was 216 minutes, and the aortic cross-clamp time was 136 minutes. She was extubated on postoperative day (POD) 9, and inotropes were weaned off on POD 10. She developed junctional ectopic tachycardia, which resolved on POD 7, as well as acute kidney injury and transaminitis, both of which improved on POD 9. She experienced a very brief episode of generalized tonic-clonic seizure, which aborted spontaneously on POD 10. Her Glasgow Coma Scale score was 15, and she was neurologically normal. A computed tomography (CT) scan of the brain was normal, and she was started on levetiracetam at 7.5mg/kg twice daily. There was presumed sepsis, which was managed with intravenous meropenem at 40mg/kg twice daily and metronidazole 10mg/kg thrice daily. The postoperative echocardiogram showed (Figures 2, 3) good ventricular function with no residual VSD, mild neo-aortic regurgitation (AR), and free-flow pulmonary regurgitation (PR). There were no ventricular outflow tract obstructions. Following a swift recovery from the complications described above, she made a good recovery and was transferred to the ward on POD 12 and discharged well on POD 23.

Operative technique

The procedure began with cardiopulmonary bypass, which was established through bicaval cannulation. The aortic cannula was positioned as high as possible in the ascending aorta, while a left ventricular vent was inserted via the right superior pulmonary vein. The proximal segment of the coronary arteries was dissected and looped for protection. After clamping the aorta, Del Nido cold blood cardioplegia, which was made up of Plasma-Lyte, lignocaine 2%, mannitol 20%, potassium chloride, magnesium sulphate, and sodium bicarbonate, was administered via the aortic root at 20mL/kg to arrest the heart.

The aorta was transected above the sinotubular junction, and the main pulmonary artery was divided just above the pulmonary valve. The branch pulmonary arteries were mobilized distally, and the ligamentum arteriosum was divided. The proximal pulmonary root was opened longitudinally into the superior aspect of the VSD. The pulmonary valve tissue was excised, and the outlet septum was divided to enlarge the VSD and to widen the left ventricular outflow tract for the aortic root. The main pulmonary artery was brought anterior to the distal ascending aorta (LeCompte maneuver) to minimize the risk of branch pulmonary artery stenosis. The aortic root was circumferentially mobilized from the right ventricular outflow tract with a rim of the right ventricular free wall. The proximal segment of the coronary arteries was carefully mobilized off the myocardium, ensuring no damage to the major coronary artery branches.

The harvested aortic root was moved posteriorly and sutured to the pulmonary annulus with a continuous polypropylene suture. The cut ends of the aorta were re-anastomosed. A tunnel was created using an acellular, pliable collagen bioscaffold patch, attaching the borders of the VSD and the anterior half of the aortic annulus. The right atrium was opened, and the patent foramen ovale was reduced using a 4mm Hegar dilator. The right atriotomy was closed, and the aortic cross-clamp was released after de-airing the heart chambers.

**Figure 4:**
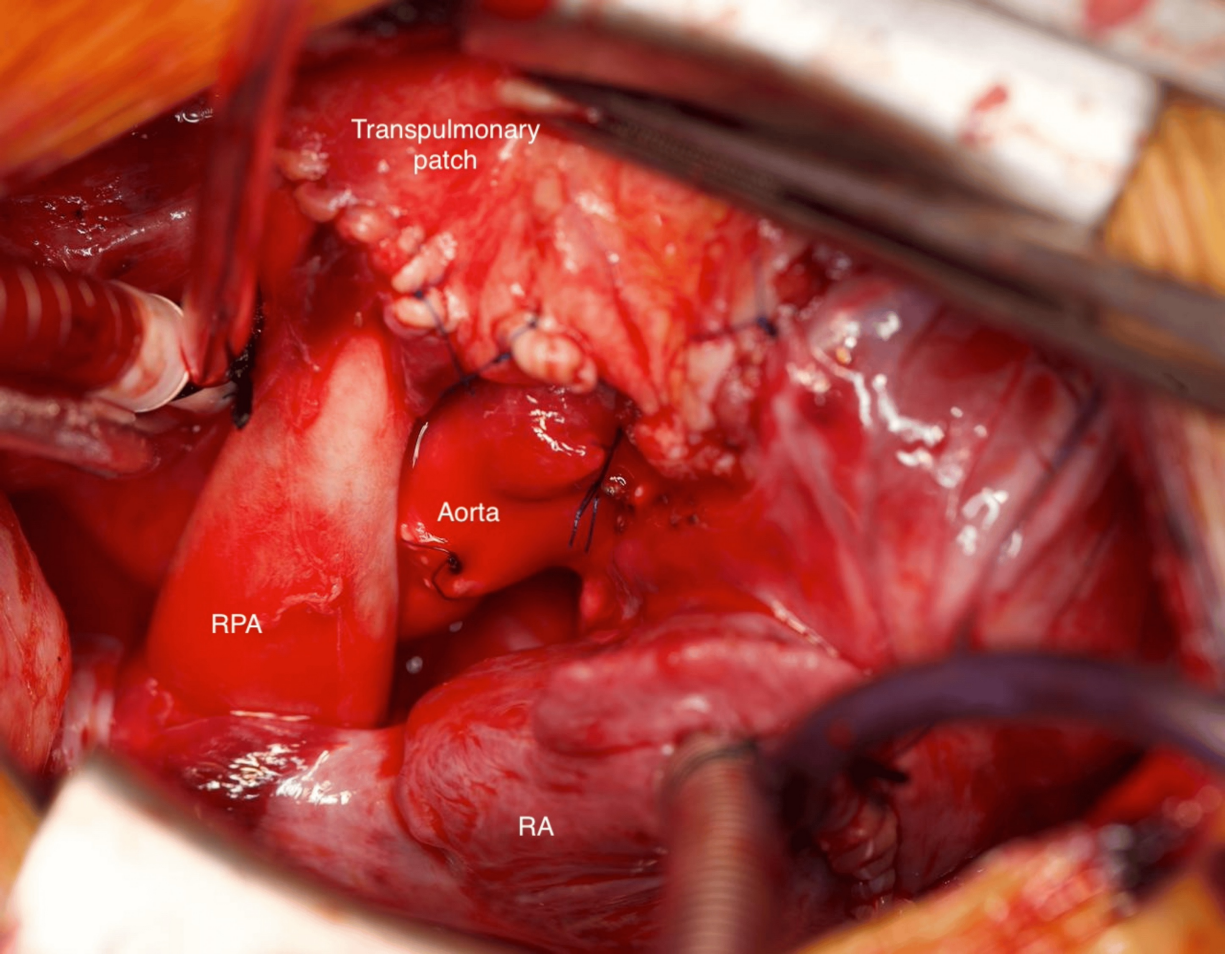
Photograph of the completed Bex-Nikaidoh procedure demonstrating the relationship of the aorta with the pulmonary artery RPA, right pulmonary artery; RA, right atrium

The right ventricle to pulmonary artery continuity was reconstructed during rewarming with the heart beating. The infundibular muscle was resected until a 12mm Hegar dilator could be inserted through the ventriculotomy. The main pulmonary artery was incised anteriorly up to the pulmonary artery confluence, and its lower free edge was sutured to the anterior wall of the ascending aorta. A large pericardial patch was used to reconstruct the right ventricular outflow tract, with the lower margin of the patch sutured to the base of the ventriculotomy, as shown in Figure 4. Cardiopulmonary bypass was terminated with the support of adrenaline and milrinone infusions, followed by modified ultrafiltration and careful hemostasis. The chest was subsequently closed in the usual manner.

## Discussion

Several surgical procedures have been proposed to address TGA with VSD and LVOTO, with each having its own advantages and limitations. The Rastelli procedure, introduced in 1969, became a standard approach for TGA, VSD, and PS [1]. This procedure involves creating an intraventricular tunnel to direct blood flow from the left ventricle to the aorta and establishing a right ventricle to pulmonary artery continuity using a valved conduit. However, the Rastelli procedure is associated with long-term complications such as recurrent LVOTO, right ventricle to pulmonary artery conduit obstruction, and arrhythmias. Kreutzer et al. reported low early mortality but substantial late mortality and morbidity associated with LVOTO, conduit obstruction, and arrhythmias [7]. There were 43% requiring reoperation and 27% requiring interventional catheterization due to conduit stenosis, whereas another 10% required reoperation due to LVOTO. Dearani et al. reported that 91% required late reoperation or intervention, of which 72% were due to conduit failure while 1% were due to LVOTO [8].

Alternative procedures, such as the REV procedure, have been developed to overcome these limitations [2]. The REV procedure involves resection of the infundibular septum and direct connection of the pulmonary trunk to the right ventricle. It creates a straight, short left ventricle to aorta tunnel and properly aligns the left ventricle with the aorta, thus eliminating the risk of late subaortic stenosis. The absence of extracardiac valved conduit reduces the need for reoperation due to recurrent pulmonary outflow tract obstruction. The REV procedure demonstrated considerable improvement over the Rastelli procedure in terms of survival and the need for reoperation for RVOTO or LVOTO. Di Carlo et al. reported that the overall survival and freedom from reoperation at 25 years for the REV procedure were 85% and 45%, respectively [9]. There were 21% cases with RVOTO and 2% cases with LVOTO who required reoperation, whereas 23% had arrhythmias. While the REV can generally achieve these promising results, it is still associated with higher early mortality and often requires revision of the RVOT.

Another alternative is the Bex-Nikaidoh procedure, which involves translocation of both the aortic and pulmonary roots [4]. This allows for a more anatomically aligned LVOT and preservation of the right ventricular cavity. The Bex-Nikaidoh procedure offers several advantages over the Rastelli procedure. The advantages include having a straight connection between the left ventricle and the aorta and it could be performed in cases with complex anatomy which are not amenable to the Rastelli or REV procedures [5].

However, the Bex-Nikaidoh procedure is technically demanding, with risks of destabilizing the aortic valve or distorting the proximal coronary arteries. Despite these challenges, the results of the Bex-Nikaidoh procedure and its modifications that were published to date are promising. The Bex-Nikaidoh procedure can be performed regardless of the VSD location and morphology and can also be performed on patients with additional intracardiac malformations, which preclude the Rastelli procedure, such as straddling of the atrioventricular valves [5]. Overall, the early mortality for the Bex-Nikaidoh procedure is low, ranging from 0% to 5%. Yeh et al. studied 19 patients who underwent the Bex-Nikaidoh procedure with a median follow-up of 11 years and reported that 26% had RVOTO requiring reoperation and none had LVOTO or aortic insufficiency requiring reoperation [10].

This case report of a child undergoing the Bex-Nikaidoh procedure exemplified its utility in managing complex cases of TGA/VSD/PS. Her specific anatomy, including the outlet VSD and severe subpulmonary obstruction, made her a less-than-ideal candidate for other procedures, highlighting the need for a more tailored surgical approach such as the Bex-Nikaidoh procedure, to achieve an optimal outcome. Continued research is needed to refine the surgical techniques and the patient selection criteria for the Bex-Nikaidoh procedure to improve its long-term outcomes in treating TGA/VSD/PS.

## Conclusions

This case demonstrated the value of tailored surgical approaches in managing complex congenital heart disease. While technically demanding, the Bex-Nikaidoh procedure remains an effective option for carefully selected patients with TGA/VSD/PS, particularly when conventional approaches carry higher risks. The Rastelli and REV procedures are alternative procedures that could be considered, but they carry higher risks of RVOTO and LVOTO that necessitate reinterventions.
